# Obstructive sleep apnea and primary snoring in children are associated with oropharyngeal dysbiosis and a mild compositional imbalance in the gastrointestinal tract

**DOI:** 10.1007/s44470-026-00058-y

**Published:** 2026-05-12

**Authors:** Jennifer Hudson, Azleena Akhand, Moe Thant Nwe, Michael J. Coffey, Josie van Dorst, Sandra Chuang, Chee Y. Ooi

**Affiliations:** 1 School of Clinical Medicine, Discipline of Pediatrics and Child Health, The University of New South Wales, Sydney, Australia; 2 Department of Gastroenterology, Sydney Children’s Hospital, Randwick, Australia; 3 Department of Respiratory Medicine, Sydney Children’s Hospital, Randwick, Australia

**Keywords:** Obstructive sleep apnea, Primary snoring, Oropharyngeal microbiome, Gastrointestinal microbiome, Pediatric

## Abstract

**Background:**

Obstructive sleep apnea (OSA) and primary snoring (PS) represent a spectrum of sleep-related breathing conditions. Emerging evidence links OSA to perturbations in the oropharyngeal and gut microbiomes and the pathogenesis of OSA-related comorbidities. However, the microbiome composition and factors driving dysbiosis in children remain unresolved.

**Methods:**

Two pediatric cohorts were examined to assess the microbiome associated with sleep-disordered breathing in the airway (Cohort A) and gut (Cohort B) using 16S rRNA gene profiling. Oropharyngeal swabs were collected from participants with OSA, PS, and healthy controls (HC) (*n* = 60). Cohort B participants (OSA and HC, *n* = 46) provided stool samples for microbiome and fecal calprotectin measurements and completed a dietary survey.

**Results:**

Oropharyngeal microbial diversity differed between OSA and HC, characterized by an increase in gastrointestinal specific taxa and reduced oral commensals. Similar shifts were observed between PS and HC, with few taxa differing between OSA and PS. In the gut, children with OSA showed an imbalance marked by an increase in opportunistic pathogens and reduced beneficial organisms. However, diversity assessments did not show any indication of dysbiosis or inflammation, and there were no overall differences in dietary intake.

**Conclusions:**

Dysbiosis in the oropharyngeal microbiomes of OSA and PS points to shared pathophysiological factors (e.g., snoring) as possible drivers of microbiome disruption across the spectrum of sleep-disordered breathing. The gastrointestinal microbiome of children with OSA indicates a mild microbial imbalance that may elicit harmful outcomes or manifest as dysbiosis if left untreated. Together, these findings support a role of the microbiome as a possible mediator of comorbidities across the spectrum of sleep-disordered breathing.

**Brief summary:**

**Current knowledge/study rationale:** Obstructive sleep apnea (OSA) and primary snoring (PS) represent a continuum of sleep-related breathing disorders. While adult and animal studies suggest OSA-induced microbiome disruptions contribute to the onset of comorbidities, the factors driving dysbiosis in children remain unresolved. This study investigated the airway and gut microbiomes in pediatric OSA, PS, and healthy controls to identify microbial alterations linked to these conditions.

**Study impact:** This study revealed that children with obstructive sleep apnea and primary snoring exhibit similar altered oropharyngeal microbiomes, distinct from that of healthy controls, suggesting common underlying pathophysiological factors.

**Supplementary Information:**

The online version contains supplementary material available at 10.1007/s44470-026-00058-y.

## Introduction

Sleep-disordered breathing (SDB) encompasses a spectrum of sleep-related breathing conditions ranging from mild primary snoring (PS) to obstructive sleep apnea (OSA). OSA, the most severe form of SDB, is a relatively common condition in children [1] typically caused by enlarged tonsils and adenoids and presents as snoring, night-time restlessness, behavioral problems, and learning difficulties. In severe cases, pediatric OSA can manifest as metabolic syndrome and hypertension—complications that can persist into adulthood if left untreated [2–4].

Across the lifespan, the occurrence of OSA is accompanied by perturbations (dysbiosis) in the oropharyngeal and intestinal microbiomes [5], coinciding with increased local and systemic inflammation [2]. Given the integral function of the microbiome as a determinant of health and disease, recent evidence now supports the role of the microbiome as a mediator of OSA-induced comorbidities [6–10].

In the upper respiratory tract, studies have described altered microbiome profiles in children and adults with OSA, compared to those without, along with an increase in the proportion of potentially pathogenic taxa such as *Neisseria* [5, 11], although results across studies report inconsistent findings [12–14]. The underlying mechanistic cause of microbial dysbiosis in the upper airway is thought to be driven by the synergistic effects of OSA-induced intermittent hypoxia, which can favor the growth of anaerobic organisms and chronic inflammation [15, 16]. However, other research has postulated that changes to the oral microbiota may also be influenced by factors such as mouth breathing, reduced salivary flow, and mechanical vibrations that are common across the spectrum of SDB, including primary snorers [14, 17]. Moreover, although the frequency of obstructive apnea/hypopnea events is the accepted marker of OSA severity, it is unclear whether the degree of oxygen desaturation serves as a more suitable predictor of microbiome disturbance [18]. As such, the specific contribution of OSA and hypoxia on the oropharyngeal microbial community of children remains unresolved.

In the gastrointestinal tract, alterations in the intestinal milieu, including disruptions to both the microbiome and intestinal barrier, have been reported. Gastrointestinal dysbiosis in adults with OSA is characterized by reduced species diversity, a higher Firmicutes:Bacteroidetes ratio (a marker of gut dysbiosis), an enrichment of taxa such as *Ruminoccocus*,* Lachnoclostridium*, and *Lachnospira* [5], and increased systemic inflammatory markers [19, 20]. Studies of animal models have further suggested that intermittent hypoxia plays a primary role in the pathogenesis of cardiovascular-related comorbidities via the action of the gut microbiota, with dietary factors contributing to these adverse outcomes [6]. Other animal studies have also implicated intermittent hypoxia from OSA in causing gut barrier dysfunction—this, in turn, facilitates gut bacterial translocation across the gut barrier into the systemic circulation, which contributes to systemic inflammation [10, 21]. It is unknown if this gut barrier dysfunction and gut dysbiosis are similarly linked with the presence of gut inflammation seen in the context of many diseases, including cystic fibrosis, COVID-19, and inflammatory bowel disease [22–24]. Very few pediatric studies evaluating the gut microbiome have been reported, with conflicting changes in microbial diversity profiles reported [25, 26].

Overall, the effects of OSA on the oropharyngeal and gut microbiota of children remain poorly defined [5, 12, 14], hindering our understanding of the mechanistic processes that lead to poorer health outcomes and further limiting the development of potential disease mitigation strategies such as microbiome modulation [9, 27]. Therefore, this study comprised two primary aims. The first aim was to characterize the oropharyngeal microbiome of children with OSA (a severe form of SDB), relative to primary snorers (a mild form of SDB) and healthy controls, and to examine its association with apnea/hypopnea frequency and oxygen desaturation during sleep. The second objective was to assess the gastrointestinal microbiome of children with moderate-severe OSA, compared to healthy controls, and to evaluate the correlation with dietary intake factors and gastrointestinal inflammation.

## Materials and methods

### Study design and participant recruitment

This study was conducted at the Sydney Children’s Hospital (SCH), Randwick, Australia, as a single-center, prospective, observational cohort study, under the “Evaluating the Alimentary and Respiratory Tracts in Health and Disease” protocol, described by Coffey et al. [28] (ethics approval number: HREC/18/SCHN/26). Children under 16 years of age reporting symptoms of sleep-disordered breathing (e.g., snoring) were recruited from sleep laboratories where they underwent overnight polysomnography. Children with an obstructive apnea/hypopnea index (OAHI) ≥ 1/h were classified as OSA and further categorized as either mild (OAHI ≥ 1/h and < 5/h) or moderate-severe (OAHI ≥ 5/h) [29]. Participants with OSA were age- and gender-matched to healthy controls and/or primary snorers. Primary snorers (PS) were children who underwent overnight polysomnography and received an OAHI < 1/h and had no other clinical conditions apart from snoring. Healthy controls (HC) were recruited from orthopedic, plastics, burns and eye clinics, who had no medical conditions apart from their presenting complaint.

Participants were excluded from this study if they had experienced an acute infection within the 4 weeks prior to sample collection, were taking regular medications that may alter the microbiome (e.g., antibiotics), those with chronic gastrointestinal conditions (e.g., inflammatory bowel disease), or if they followed specific dietary regimes (e.g., gluten free).

**Fig. 1:**
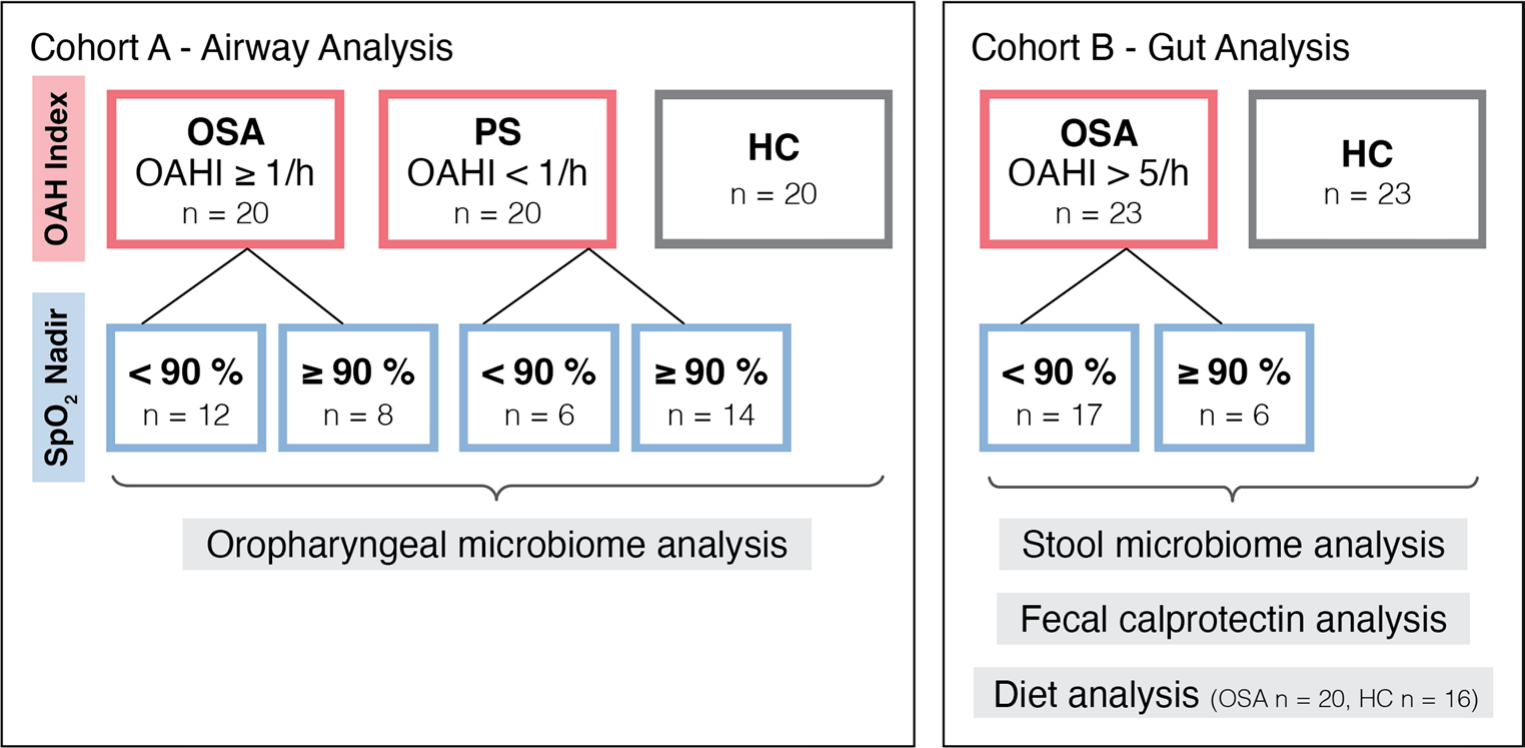
Study design and sample size of Cohort A for oropharyngeal microbiome analysis and Cohort B for stool microbiome, calprotectin, and diet analysis

This study comprised two separate cohorts, recruited between 2018 and 2020. Cohort A consisted of OSA (OAHI ≥ 1/h), PS, and HC participants and was recruited for the purpose of investigating the effect of obstructive sleep-disordered breathing on the oropharyngeal microbiome. Cohort B consisted of moderate-severe OSA (OAHI > 5/h) and HC and was recruited for the purpose of investigating the effect of OSA on the gastrointestinal microbiome (Fig. 1).

For an analysis of the impact of hypoxia on the microbiome, OSA and PS from each respective cohort were regrouped based on the lowest recoded oxygen saturation (SpO_2_ nadir): SpO_2_ < 90% (SpO_2_ nadir low) and SpO_2_ nadir ≥ 90% (SpO_2_ nadir high). These groups were compared to HC (Fig. 1).

### Sample collection

Following consent, oropharyngeal swabs were collected from Cohort A participants on the day of the sleep study and stored at − 80 °C until further use. Stool samples from Cohort B were mailed to the laboratory in an insulated cooler with an icepack and stored at − 80 °C upon receipt.

Parents of participants in Cohort B were additionally requested to complete the validated Australian Children and Adolescent Eating Survey, based on the previous 6 months of food intake, to determine macro- and micronutrient intake [30].

### Microbiome analysis

Genomic DNA was isolated from swabs and stool using the QIAamp DNA Blood Mini Kit and QIAamp Fast DNA Stool Mini Kit (Qiagen), respectively, according to the manufacturer’s instructions. Amplification of the V4 region of the 16S rRNA gene by PCR was performed with primers 515 F (GTGYCAGCMGCCGCGGTA) and 806R (GGACTACNVGGGTWTCTAAT). The amplified DNA was sequenced on the Illumina MiSeq Platform at the Ramaciotti Centre for Genomics at UNSW Sydney.

Quality filtering was performed according to the thresholds outlined in Coffey et al. [31]. Processed sequences were clustered in unique sequences (zero-distance operational taxonomic unit (zOTU)) with the unoise2 algorithm implemented in USEARCH [32]. Chimeric sequences were first removed *de novo *during the UNOISE processing step. A subsequent reference-based chimera check was performed using the UCHIME algorithm against the SILVA 123 database [33]. After chimera removal, sequences were classified by BLASTn alignment against the SILVA database. Concatenated sequences of all sequences were mapped on the final set of zOTUs to calculate the abundance of each zOTU for each sample. Non-bacterial sequences were removed.

Alpha-diversity (a metric of species richness and evenness within individual samples) was measured using the Shannon–Weaver index with statistical differences between OAHI-based grouping (i.e., OSA vs PS vs HC) and SpO_2_ nadir (i.e., < 90% vs ≥ 90% vs HC) groups determined using a one-way analysis of variance (ANOVA). Differences between samples were calculated from square-root transformed count data using Bray–Curtis dissimilarity indices. Differences between groups were determined using a permutational analysis of variance (PERMANOVA) with 999 permutations using the “adonis2” function, with pairwise comparisons performed using the “pairwiseAdonis” (v0.4.1) function in the “vegan” package (v2.6.10).

Differential abundance analysis was performed using the “Analysis of Compositions of Microbiomes with Bias Correction 2” (ANCOM-BC2) package (v2.9.2) to assess differences in taxonomic composition between groups, using default parameters. Taxa with a false discovery rate (FDR) < 0.05 and a log fold change (LFC) > 1 were considered to be differentially abundant.

### Calprotectin measurement

Fecal calprotectin (a marker of intestinal inflammation) was extracted and quantified using the fCAL enzyme-linked immunosorbent assay (ELISA) kit (Buhlmann), following the manufacturer’s instructions. Samples exceeding the assay range were appropriately diluted and re-analyzed. Samples with fecal calprotectin concentrations > 80 µg/g were considered abnormal, as specified by the manufacturer.

### Statistical analysis

Statistical analyses were conducted using RStudio (v4.5.0). Categorical variables were compared using either a chi-square test or Fisher’s exact test. Continuous variables were assessed for normality and compared using appropriate parametric (Student’s *t*-test or ANOVA) or non-parametric tests (Wilcoxon rank sum test or Kruskal–Wallis test). Multivariate differences in dietary intake profiles between groups were assessed using PERMANOVA via the “adonis2” function from the “vegan” package, based on a Euclidean distance matrix computed from scaled nutrient variables, with 999 permutations. All data visualization was performed using the ggplot2 package.

## Results

### General demographics

Cohort A (airway analysis) consisted of 20 OSA children that were age- and gender-matched to PS and HC, with a median age of 4.6, 5.2, and 5.2 years, respectively (Table 1). Among the OSA and PS groups of Cohort A, 18 (45%) participants recorded an oxygen saturation nadir < 90% (SpO_2_ low), with the remaining 22 (55%) classified as SpO_2_ nadir ≥ 90% (SpO_2_ high) (Fig. 1, Table 2). Whilst there was a difference in oxygen saturation nadir between the OSA and PS groups (*p* < 0.01), no differences in transcutaneous carbon dioxide peak, %REM sleep, or arousal index were detected between the groups in Cohort A (Table 2). Cohort B (gut analysis) included 23 moderate-severe OSA children and 23 age- and gender-matched HC, with a median age of 4.1 and 3.8 years, respectively. Within the OSA group, 17 (74%) recorded a SpO_2_ nadir < 90% (Fig. 1, Table 2). No differences in height, weight, or BMI z-scores were observed between groups across either cohort (*p* > 0.05) (Table 1).

**Table 1:** Demographic characteristics of Cohorts A and B

	Healthy control	Primary snorer	Obstructive sleep apnea	*p*-value
Cohort A (airway)				
Participants, *n*	20	20	20	-
Age, years	5.2 (3.5, 11.2)	5.2 (3.4, 9.5)	4.6 (2.9, 10.5)	*p* = 0.88
Male, *n* (%)	12 (60)	12 (60)	12 (60)	*p* = 1.00
Height z-score	0.4 (0.1, 1.0)	0.2 (− 0.5, 0.6)	0.4 (− 0.4, 1.0)	*p* = 0.61
Weight z-score	0.4 (− 0.1, 1.0)	0.2 (− 0.3, 0.7)	0.5 (− 0.1, 2.3)	*p* = 0.44
Body mass index z-score	0.4 (− 0.7, 0.8)	0.4 (− 0.2, 0.9)	0.8 (− 0.4, 2.2)	*p* = 0.13
Cohort B (gut)				
Participants, *n*	23	-	23	-
Age (years)	4.1 (2.8, 6.7)	-	3.8 (2.6, 5.8)	*p* = 0.81
Male, *n* (%)	16 (70)	-	16 (70)	*p* = 1.00
Height z-score	0.5 (− 0.2, 0.8)	-	0.2 (− 0.4, 0.8)	*p* = 0.44
Weight z-score	0.0 (− 0.3, 0.6)	-	0.2 (− 0.2, 1.1)	*p* = 0.42
Body mass index z-score	− 0.3 (− 0.9, 0.3)	-	0.4 (− 0.6, 1.1)	*p* = 0.10

Data presented as median (interquartile range) unless otherwise indicated

“-” denotes “not applicable”

**Table 2:** Polysomnography metrics for obstructive sleep apnea and primary snorer participants across Cohorts A and B

	Primary snorer	Obstructive sleep apnea	*p*-value
Cohort A (airway)			
OAHI (events/h)	0.1 (0.0, 0.1)	4.2 (3.1, 9.1)	*p* < 0.01
AHI (events/h)	1.1 (0.5, 1.6)	6.3 (4.0, 10.1)	*p* < 0.01
O_2_ saturation nadir (%)	91.0 (88.0, 92.3)	87.5 (80.0, 91.0)	*p* = 0.01
TcCO_2_ peak, mmHg	47.4 (43.3, 49.9)	48.6 (46.4, 53.6)	*p* = 0.12
Sleep efficiency (%)	88.4 (81.9, 91.2)	87.1 (79.1, 89.6)	*p* = 0.39
% REM sleep	19.0 (15.4, 21.8)	19.7 (17.9, 23.6)	*p* = 0.49
% NREM sleep	81.1 (78.2, 84.6)	80.4 (76.4, 82.2)	*p* = 0.49
Arousal index (events/h)	7.5 (5.2, 10.0)	8.3 (4.9, 11.9)	*p* = 0.50
Cohort B (gut)			
OAHI (events/h)	-	10.4 (8.3, 16.9)	-
AHI (events/h)	-	10.4 (8.4, 16.9)	
O_2_ saturation nadir (%)	-	82.0 (72.5, 89.5)	-
TcCO_2_ peak, mmHg	-	51.6 (47.4, 59.8)	-
Sleep efficiency (%)	-	88.5 (84.4, 91.8)	-
% REM sleep	-	19.1 (16.2, 25.7)	-
% NREM sleep	-	80.6 (73.4, 83.2)	-
Arousal index (events/h)	-	9.9 (5.8, 16.3)	-

Data presented as median (interquartile range)

“-” denotes “not applicable”

### Oropharyngeal microbiome (airway) analysis (Cohort A)

**Fig. 2:**
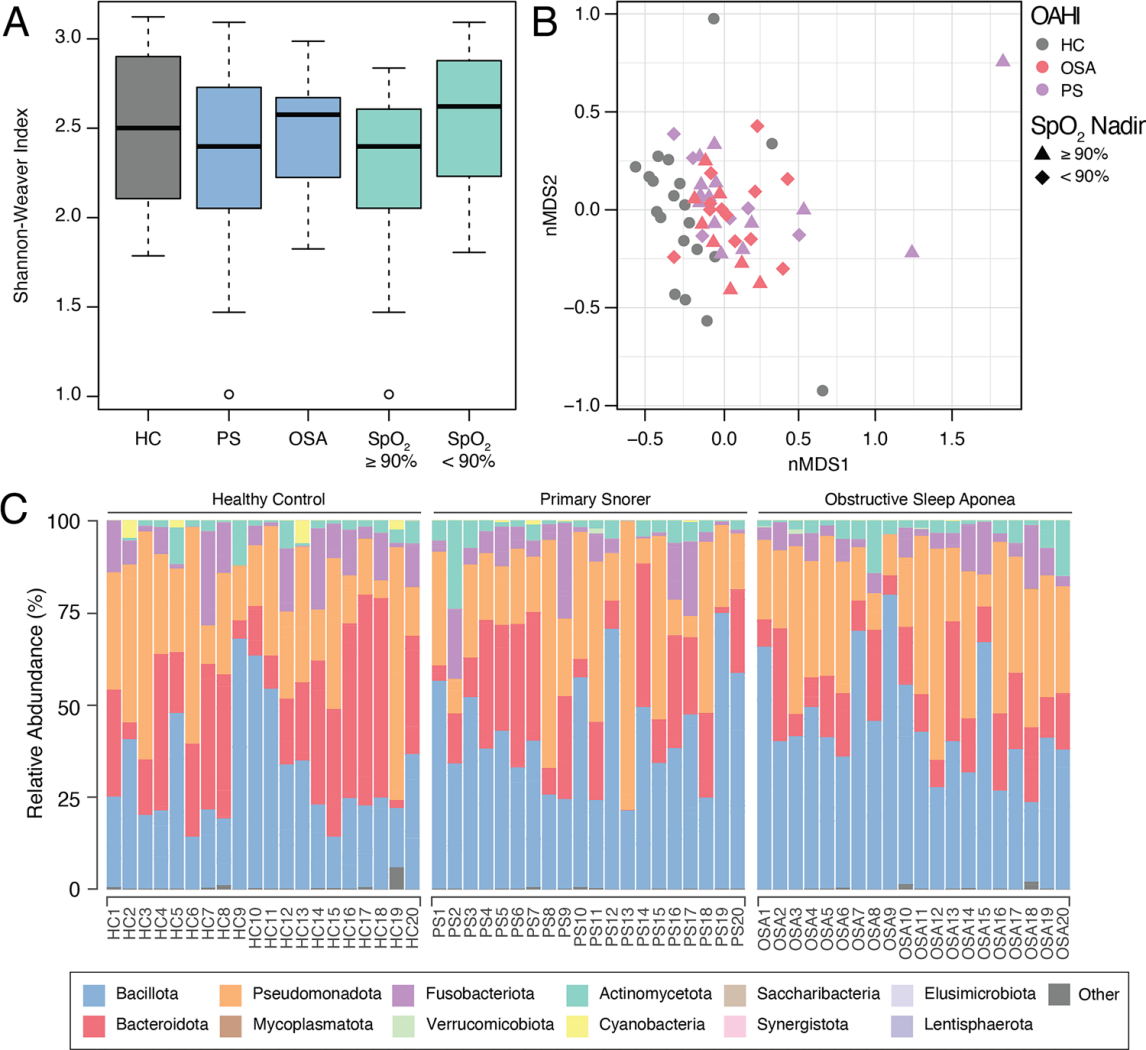
Oropharyngeal microbiome (airway) analysis (Cohort A). **A** Shannon–Weaver diversity index (alpha-diversity) of oropharyngeal microbiome based on classification by obstructive-apnea hypopnea index (blue) and oxygen saturation nadir below 90% (green). No differences were observed between HC vs PS vs OSA comparisons (*p* = 0.49) or between HC vs SpO_2_ nadir < 90% vs SpO_2_ nadir ≥ 90% comparisons (*p* = 0.25). **B** Non-metric multidimensional scaling (nMDS) plot of Bray–Curtis distances (beta-diversity) for samples classed by OAHI and hypoxia indices. Each sample is represented by a point, with points closer together reflecting samples with more similar microbiome profiles. Differences were observed between HC vs OSA (*p* < 0.01), HC vs PS (*p* = 0.03) as well as HC vs SpO_2_ nadir < 90% (*p* = 0.01) and HC vs SpO_2_ nadir ≥ 90% (*p* = 0.02). No difference between OSA and PS groups (*p* = 1.00) or SpO_2_ nadir < 90% vs SpO_2_ nadir ≥ 90% (*p* = 1.00). **C** Relative abundance plot of bacterial phyla across HC, PS, and OSA samples

After quality filtering, we identified 963 zOTUs across 60 oropharyngeal swab samples using 16S rRNA gene amplicon sequencing. The oropharyngeal microbiome was dominated by OTUs assigned to phyla Bacillota, Bacteroidota, and Pseudomonadota (Fig. 2C) and genera *Streptococcus*, *Actinobacillus*, *Prevotella*, *Veillonella*, and *Neisseria* (Figure S1). No differences in alpha-diversity, as measured by the Shannon–Weaver diversity index, were observed across Cohort A when comparing OSA, PS, and HC groups (2.6 (2.3–2.7) v 2.4 (2.0–2.7) v 2.5 (2.1–2.9), respectively, *p* = 0.49) or when comparing SpO_2_ nadir low, SpO_2_ nadir high, and HC (2.6 (2.2–2.8) v 2.4 (2.1–2.6) v 2.5 (2.1–2.9), respectively, *p* = 0.25) (Fig. 2A). Analysis of beta-diversity revealed a separation between the oropharyngeal microbiomes of healthy controls when compared to both OSA (*p* < 0.01) and PS children (*p* = 0.03), as well as against SpO_2_ low (*p* = 0.01) and SpO_2_ high (*p* = 0.02) subgroups. However, there was no difference between OSA and PS groups (*p* = 1.00) or between SpO_2_ low and SpO_2_ high groups (*p* = 1.00) (Fig. 2B).

**Fig. 3:**
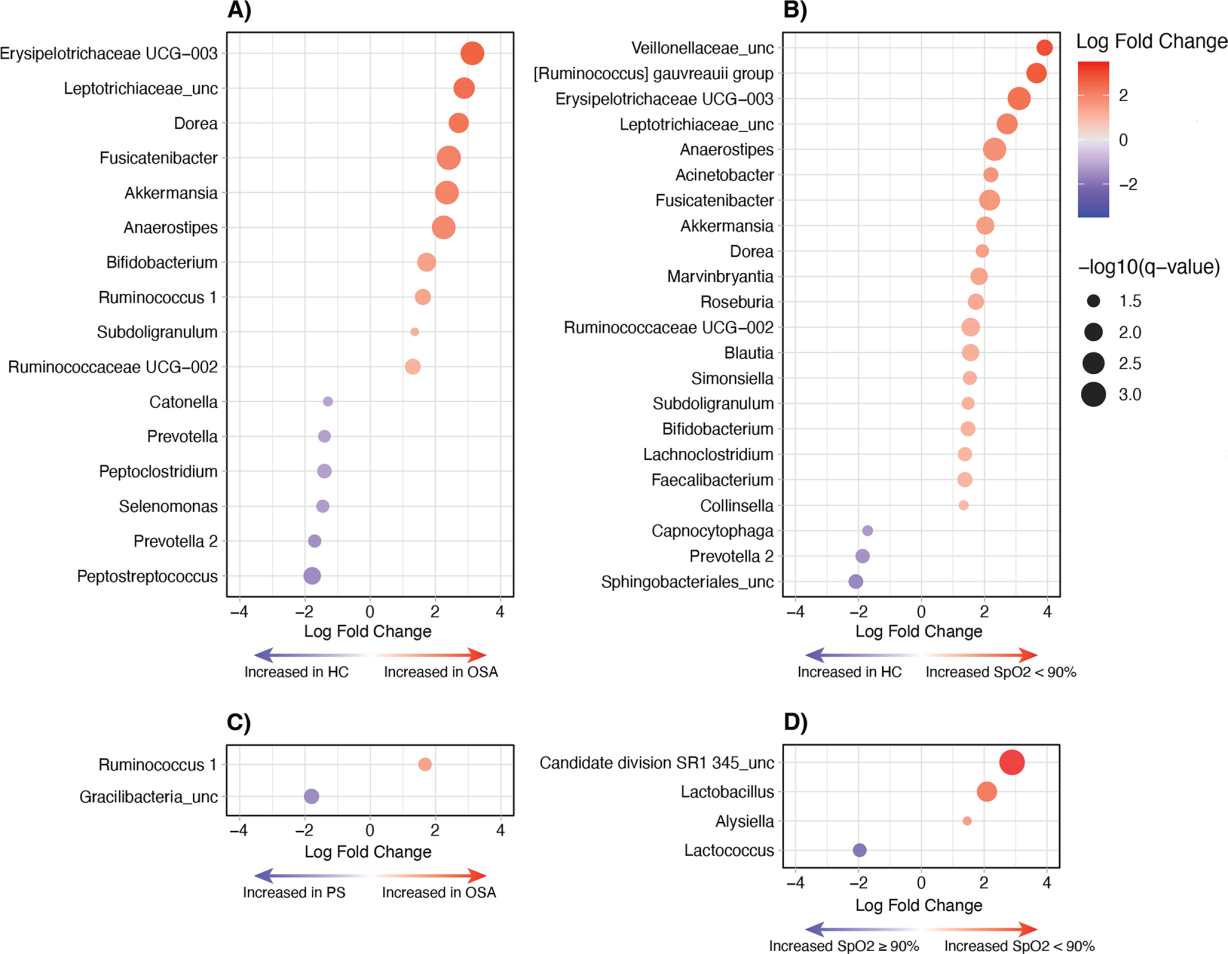
Genus level differential abundance analysis oropharyngeal microbiome (Cohort A) comparing **A** OSA to HC, **B** SpO_2_ nadir < 90% vs HC, **C** OSA vs PS, and **D** SpO_2_ nadir < 90% vs SpO_2_ nadir ≥ 90%. Only taxa with an FDR < 0.05 are displayed

At the genus level, 16 taxa were differentially abundant between OSA and HC groups, with Erysipelotrichaceae UCG-003 exhibiting the highest increase in OSA (LFC = 3.1), followed by Leptotrichiaceae and *Dorea*, with *Peptostreptococcus* showing the greatest decrease in relative abundance in OSA (LFC = − 1.8) (Fig. 3A). Eighteen taxa were differentially abundant between the PS and HC groups (Figure S2), with Erysipelotrichaceae UCG-003 also exhibiting the highest increase in PS (LFC = 2.5). When comparing OSA and PS groups, there was no significant difference between these two groups with exception of only two taxa, *Ruminococcus* 1 and an unclassified *Gracilibacteria* (Fig. 3C).

The differential abundance profile of the SpO_2_ low group, compared to HC, resembled that of the OSA-HC comparison (Fig. 3), with additional taxa, including unclassified Veillonellaceae, *Ruminococcus gauvreauii*, and *Acinetobacter*, increased in relative abundance in response to low oxygen conditions. Only the relative abundance of unclassified Sphingobacteriales, *Prevotella* 2, and *Capnocytophaga* was reduced in the SpO_2_ low-HC comparison.

### Gastrointestinal microbiome analysis (Cohort B)

**Fig. 4:**
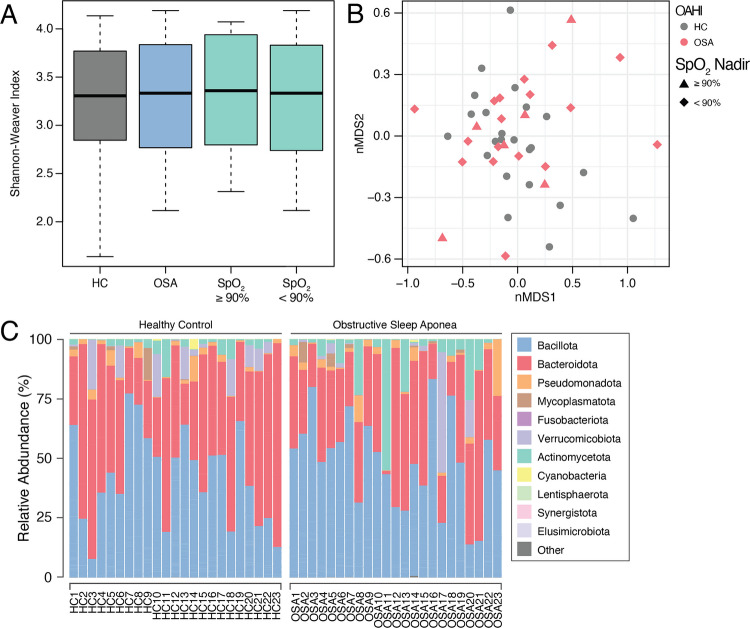
**A** Shannon–Weaver diversity index (alpha-diversity) of gut microbiome (Cohort B) samples based on classification by obstructive-apnea hypopnea index (blue) and lowest oxygen saturation below 90% (green). No differences were observed between HC and OSA (*p* = 0.78) or between HC vs SpO_2_ nadir < 90% vs SpO_2_ nadir ≥ 90% comparisons (*p* = 0.95). **B** Non-metric multidimensional scaling (nMDS) plot of Bray–Curtis distances (beta-diversity) for samples classed by OAHI and hypoxia indices. No differences were observed between HC vs OSA (*p* = 0.21) or between HC and hypoxia indices (*p* = 0.47). **C** Relative abundance plot of bacterial phyla across HC and OSA samples

**Fig. 5:**
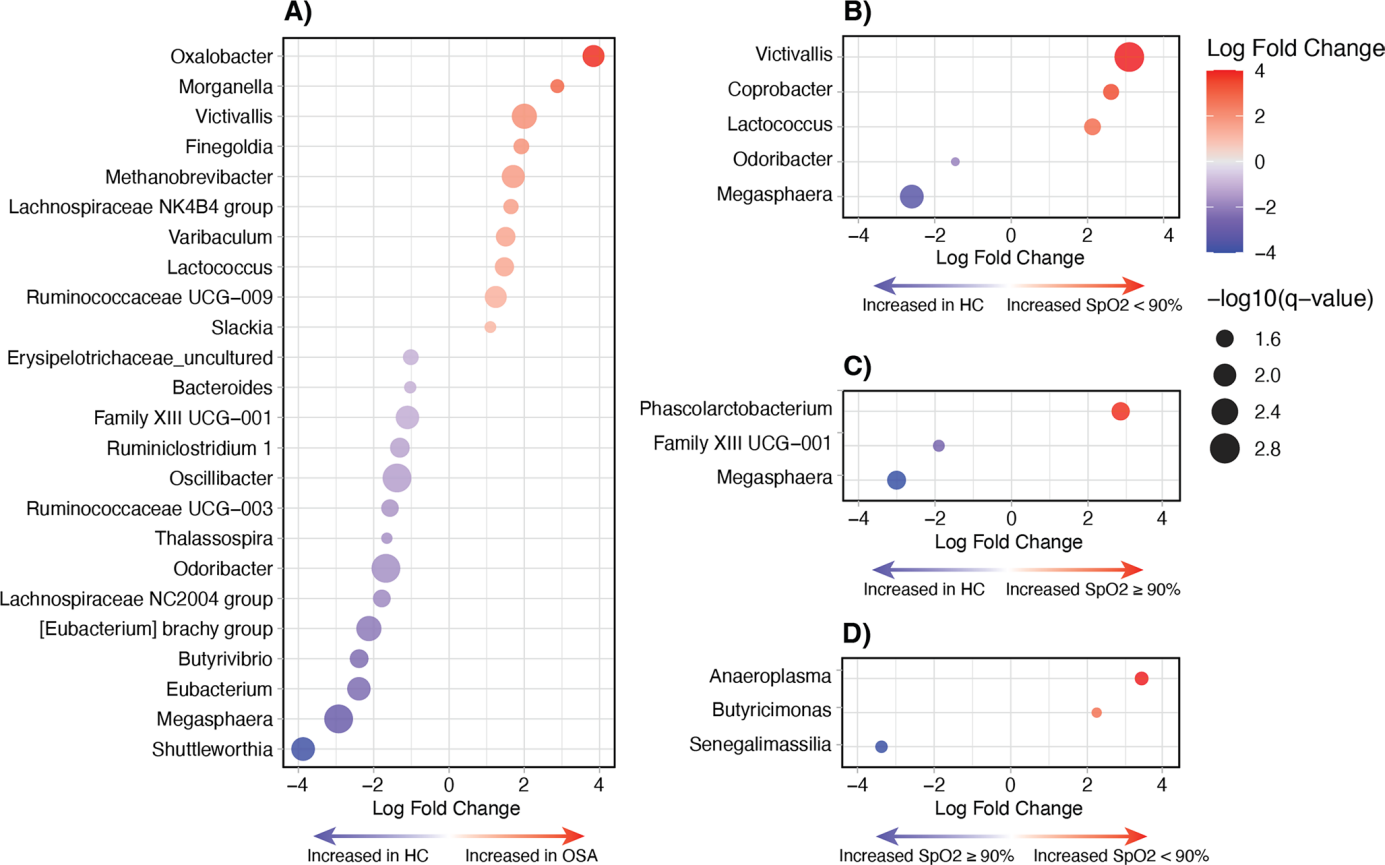
Genus level differential abundance analysis of the gut microbiome (Cohort B) comparing **A** OSA to HC, **B** SpO_2_ nadir < 90% vs HC, **C** SpO_2_ nadir ≥ 90% vs HC, and **D** SpO_2_ < 90% vs SpO_2_ ≥ 90%. Only taxa with an FDR < 0.05 are displayed

16S rRNA gene community analysis of 46 stool samples collected from the OSA and HC participants of Cohort B yielded 4,793,629 total reads which, after filtering, clustered into 1087 zOTUs. At the phylum level, the gut microbiome largely comprised members of Bacillota and Bacteroidota (Fig. 4C), with genus-level taxonomy predominantly assigned to *Bacteroides*,* Fecalibacterium*,* Alistipes*, *Akkermansia*, and *Bifidobacterium* (Figure S1). There was no distinction in the gut microbiome of OSA and HC, on the basis of alpha (3.3 (2.8–3.8) and (3.3 (2.8–3.8), respectively, *p* = 0.78) or beta (*p* = 0.21) diversity measures. Similarly, no differences were observed with respect to alpha- and beta-diversity when grouped based on SpO_2_ nadir indices (*p* = 0.95 and *p* = 0.47, respectively) (Fig. 4). Differential abundance analysis using ANCOM-BC2 revealed ten taxa were increased in abundance, whilst 14 taxa were reduced in OSA compared to HC (Fig. 5). *Oxalobacter* displayed a 3.8 log fold increase in the OSA group when compared to HC, followed by *Morganella*, *Victivallis*, and *Finegoldia*. In contrast, genus *Shuttleworthia* exhibited the largest decrease (LFC = − 3.9), followed by *Megasphaera*, *Eubacterium*, and *Butyrivibrio*. Differences were also seen when making comparisons based on SpO_2_ nadir groups, with the abundance of five taxa distinguished between SpO_2_ low group and HC, and only three taxa between SpO_2_ low (*n* = 17) and SpO_2_ high (*n* = 6) subgroups (Fig. 5). No difference was observed in the Firmicutes:Bacteroidetes ratio between OSA and HC groups (1.4 (1.0–2.1), 1.0 (0.4–2.0), respectively, *p* = 0.14) (Figure S3).

### Calprotectin analysis

The median fecal calprotectin concentration of OSA and HC participants of Cohort B was 43.0 (28.4–131.0) µg/g and 66.7 (19.1–113.7) µg/g, respectively (Figure S3) with both median values falling within the range considered normal (< 80 µg/g, Bulhmann). No statistically significant difference was observed between the groups (*p* = 0.63).

### Diet analysis

Multivariate analysis of dietary intake data (normalized to per 1000 kJ) did not show any overall differences in dietary composition between OSA and HC (*p* = 0.26). However, pairwise analyses between individual nutrient types revealed that the OSA group had reduced dietary intake of total folates, niacin, and potassium (*p* < 0.05) (Figure S4).

## Discussion

This is the first study to demonstrate that children across the spectrum of obstructive sleep-disordered breathing, ranging from PS to OSA, exhibit microbial perturbations in the oropharyngeal cavity when compared to healthy controls, with no major distinction between the frequency of obstructive events or the degree of oxygen desaturation. In contrast, the gastrointestinal microbiomes did not reveal any community-level differences between moderate-severe OSA and HC but did demonstrate mild compositional imbalances.

In Cohort A, beta diversity analysis revealed a deviation in the oropharyngeal microbiome of those with OSA from healthy controls, indicative of dysbiosis. Pairwise comparisons revealed that differences in the microbiome of those with OSA, when compared to HC, were due to an enrichment of organisms usually confined to the gastrointestinal tract, including Erysipelotrichaceae, *Dorea*, *Fusicatenibacter*, *Akkermansia*, *Anaerostipes*, *Ruminococcus*, and *Subdoligranulum*, and a reduction of oral commensals (Fig. 3A). The enrichment of gut-specific microorganisms in the oral environment is not commonly reported in adult or pediatric OSA studies [13] but has been described in the oral and esophageal microbiomes of individuals with reflux disease [34, 35], with accumulating evidence linking OSA and gastroesophageal reflux conditions [36]. Together, the translocation of gut-specific taxa to the mouth, in conjunction with reduced oral commensals, may promote a state of dysbiosis leading to the onset of oral health-related comorbidities (such as caries and periodontal disease) [37–39], with potential implications for broader systemic health [40].

Although OAHI is utilized as the standard diagnostic marker of OSA, the degree of oxygen desaturation has been postulated as an alternative indicator of the physiological impact of obstructive sleep-disordered breathing. In this study, the OSA group did exhibit a lower median oxygen saturation nadir than PS (Table 2); however, 30% of PS participants from Cohort A (airway) recorded an SpO_2_ nadir < 90%, whilst 40% of OSA had an SpO_2_ nadir ≥ 90% (Fig. 1). Regardless, similar differential abundance profiles were obtained when comparing either the SpO_2_ nadir low (< 90%) or abnormal OAHI groups to the healthy control group (Fig. 3). The SpO_2_ nadir low group, when compared to HC, was comprised of an increased abundance of anaerobic taxa (Fig. 3B), which included an enrichment of unclassified species from Veillonellaceae, *Ruminococcus gauvreauii* group, and *Marvinbryantia*, as well as *Acinetobacter*. Veillonellaceae, particularly genus *Veillonella*, are considered core commensal members of the oral microbiome where they can confer beneficial properties in a healthy microbiome [41], but are recognized as “accessory pathogens” in dysbiosis states associated with periodontal disease [42]. Likewise, members of *Acinetobacter*, including *Acinetobacter baumannii*, are opportunistic pathogens associated with periodontitis and other mucosal infections, further supporting the potential microbial links between OSA- and dental-related comorbidities [14, 43].

Interestingly, the oropharyngeal microbiome of children with an OAHI < 1/h, classified as primary snorers (PS), was also distinct from that of the HC group, aligning more closely to those diagnosed with OSA (Fig. 2B). A similar finding was also seen when comparing based on oxygen desaturation metrics, with the microbiome of those with a high SpO_2_ nadir (≥ 90%) also resembling that of the low SpO_2_ nadir (< 90%) group. Additionally, pairwise comparisons showed that only a few taxa differed in relative abundance between PS and OSA, or between high and low oxygen saturation nadir (Fig. 3C and D), with these limited differences unlikely to elicit any OSA-specific comorbidities. Therefore, dysbiosis in the oropharyngeal microbiome of children with OSA may primarily be driven by pathophysiological factors common to PS (such as mouth breathing, reduced salivary flow, and mechanical vibrations) rather than the frequency of apnea/hypopnea events or hypoxia severity. Furthermore, studies have argued that primary snoring in children, in the absence of frequent apnea/hypopnea events, is not inherently benign and can manifest as similar clinical outcomes as those with OSA, including hypertension [44, 45] and neurocognitive deficits [46, 47]. Given the similarities between the oropharyngeal microbiomes in our cohort of OSA and PS, relative to HC, oropharyngeal dysbiosis may arise across the full spectrum of obstructive sleep-disordered breathing, due to shared underlying pathophysiology, and contribute to a wider occurrence of comorbidities across the spectrum of sleep-disordered breathing. Further microbiome studies in children are required in the future to fully elucidate this relationship.

The gastrointestinal microbiome of children with OSA exhibited a similar diversity profile to that of age- and gender-matched healthy controls, with no differences observed with respect to alpha- and beta-diversity measures (Fig. 4) and no changes to the Firmicutes:Bacteroidetes ratio (Figure S3), thus providing no evidence of gut dysbiosis in this cohort. Although these findings contrast with the diversity metrics described in the few reported pediatric OSA studies, these discrepancies may arise from differences in participant age range, the method used to assess diversity (i.e., ANOSIM vs PERMANOVA) [25] and small sample size [26].

Although no community-level differences were observed, pairwise comparisons between the gut microbiome of those with OSA and HC did reveal an increase in the abundance of potentially pathogenic taxa and a reduction in beneficial genera. The ten taxa increased in the OSA group (Fig. 5) included *Finegoldia* and *Morganella*, which have been associated with a range of infections or inflammatory conditions [48–51]. Lachnospiraceae and Ruminococcaceae, both of which were increased in abundance, have also been found to be enriched in the gut microbiome of adults with OSA-induced hypertension [52]. Of the 14 taxa reduced in abundance in OSA, the majority are short-chain fatty acid (SCFA) producers, including *Butyrivibrio*, *Odoribacter*, *Oscillibacter*, *Bacteroides*, and *Megasphaera* [53, 54] (Fig. 5). In OSA, a reduced abundance of SCFA-producing microorganisms in the gut has been described and postulated as a causal factor of increased hypertension- and obesity-related comorbidities in adults and animal studies [7, 19, 25]. Further investigation is required to determine whether these mild alterations can be attributed to the onset and progression of comorbidities associated with pediatric OSA. Alternatively, these imbalances may signify early stages of dysbiosis with the potential to cause adverse outcomes if SDB is left untreated, but further longitudinal data are needed to disentangle this relationship.

In addition to their potential role in the development of OSA-related comorbidities, SCFAs also confer broader beneficial functions in the gut by maintaining barrier integrity and positively mediating intestinal inflammation [55]. However, this study found no evidence for gastrointestinal inflammation as measured by fecal calprotectin in children with OSA (Figure S3), suggesting that these alterations to the intestinal microbiome profile of OSA children are not sufficient to elicit a localized inflammatory response. Other studies have reported elevated systemic inflammatory markers in children with OSA [19, 56], but gastrointestinal inflammation, as measured by fecal calprotectin, has not yet been evaluated.

The occurrence and severity of OSA are often positively correlated with an elevated BMI, especially in the adult population, which in turn can further exacerbate disease severity [57]. However, in Cohort B, there was no difference between the median BMI z-score of children with OSA compared to HC (Table 1). The relationship between BMI and OSA is often attributed to diet, as reduced sleep quality may promote daytime fatigue, hormone disruption, and insulin resistance, ultimately manifesting as increased appetite [58, 59]. Indeed, some studies have indicated poorer dietary habits in children with OSA, such as increased sugar and fast food consumption [59]. However, no differences in the dietary intake profile between OSA and HC were observed in this study, nor was there any difference in the consumption of sugar, fat, protein, or carbohydrates (Figure S4). Pairwise analyses did find that the OSA group reported reductions in their dietary intake of folate, niacin, and potassium (Figure S4). These essential vitamins and minerals are critical for both host and microbial physiological functioning and may be associated with some of the variations observed in the gut microbiome.

Overall, this study is strengthened by its carefully designed methodology. Current understandings of the relationship between pediatric OSA and the microbiome are predominantly based on comparisons to a HC cohort. Here, the inclusion of a PS comparison, in addition to a HC group, allowed for a more comprehensive insight into the broader impact of SDB on the microbiota. Age- and gender-matching across all groups reduced the influence of age-related variations and strengthens the interpretation of OSA-specific microbial changes. Although no community-level alterations in the gut microbiota were observed, the inclusion of only moderate-severe cases of OSA ensured that any clinically relevant differences (if present) would be detected. With regard to limitations, the results of this study are constrained by the small sample size, obtained from a single center at a single point in time. Furthermore, whilst 16S rRNA gene sequencing is a well-established method for microbial community profiling and the detection of compositional changes, it is unable to provide species-level resolution or functional insights. Accordingly, future studies employing functional-based approaches (such as metagenomic sequencing) are needed to broaden our understanding of dysbiosis in the airway of children with OSA and PS.

## Conclusions

The findings of this study demonstrate that children with obstructive sleep-disordered breathing ranging from primary snorer to OSA exhibit an altered oropharyngeal microbiome profile compared to that of healthy controls. The disruption in the oropharyngeal microbiome profile does not seem to differentiate based on the frequency of obstructive events (OAHI) or degree of hypoxia during sleep, suggesting that oropharyngeal microbiome dysbiosis is related to common pathophysiological factors that underlie the full spectrum of obstructive sleep-disordered breathing, and that primary snoring may not be benign. In the context of the gut microbiota, our cohort of moderate-severe OSA children did not demonstrate community level differences, nor was there evidence linking the gut microbiome as a causal factor of OSA-related comorbidities through nutritional intake and intestinal inflammatory pathways. However, a reduction in beneficial taxa and an increase in opportunistic pathogens suggest a subtle microbiome imbalance, potentially linked to the onset of pediatric OSA comorbidities. Further studies investigating the microbiome of pediatric OSA within the broader context of sleep-disordered breathing may provide deeper insight into the underlying mechanisms influencing disease progression and systemic health.

## Supplementary Information

Below is the link to the electronic supplementary material.

Supplementary file1 (DOCX 10.2 MB)

## Data Availability

Sequencing data has been deposited in the NCBI Sequence Read Archive (SRA) under the BioProject PRJNA1312271.
