# Supplementary material for: Obstructive sleep apnea and primary snoring in children are associated with oropharyngeal dysbiosis and a mild compositional imbalance in the gastrointestinal tract
Source: J Clin Sleep Med. 2026 May 12;22(1):76. doi: 10.1007/s44470-026-00058-y (PMC13168413; doi:10.1007/s44470-026-00058-y)
Supplement: Supplementary file 1 — Supplementary file1 (DOCX 10.2 MB) [file 44470_2026_58_MOESM1_ESM.docx]

**Supplementary file**

**Obstructive sleep apnea and primary snoring in children is associated with oropharyngeal dysbiosis and a mild compositional imbalance in the gastrointestinal tract**

Jennifer Hudson^1,#^, Azleena Akand^1^, Moe Thant Nwe^1^, Michael J. Coffey^1,2^, Josie van Dorst^1^, Sandra Chuang^1,3,*^ and Chee Y. Ooi^1,2,*^

^1^School of Clinical Medicine, Discipline of Pediatrics and Child Health, The University of New South Wales, Sydney, Australia.

^2^Department of Gastroenterology, Sydney Children’s Hospital, Randwick, Australia.

^3^Department of Respiratory Medicine, Sydney Children’s Hospital, Randwick, Australia.

*Joint Senior Authors

^#^Corresponding Author: jennifer.hudson@unsw.edu.au

**Table of Contents**

**Supplementary Figure S1**: …….….….………………………………………………………**3**

**Supplementary Figure S2**: …………………….……………………..………………………**4**

**Supplementary Figure S3:** ……………….………………………………..…………………**5**

**Supplementary Figure S4:** ……………….………………………………..…………………**6**


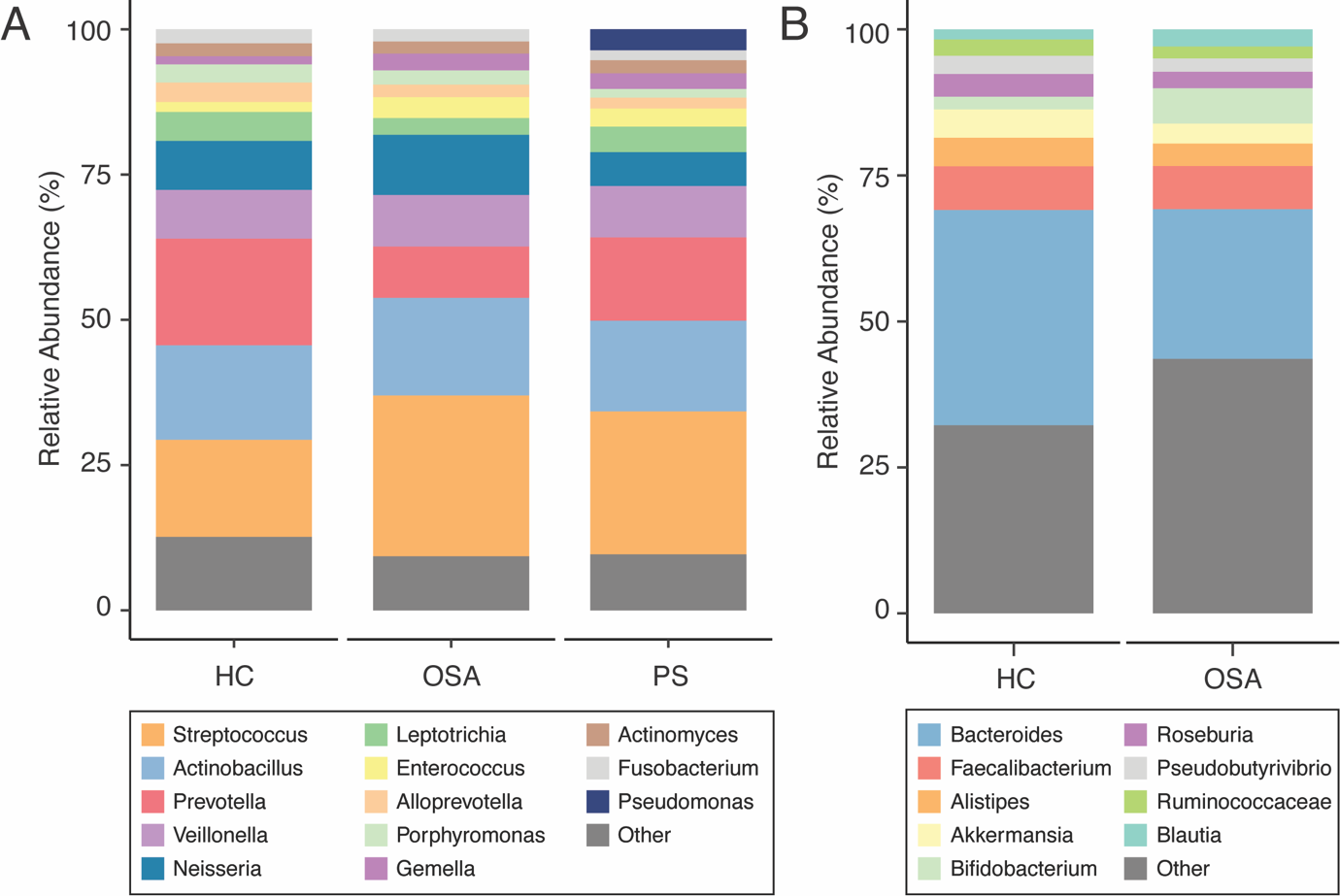


Supplementary Figure S1: Mean relative abundance of genus level taxonomic assignment of the oropharyngeal (A) and gut (B) microbiomes. Only taxa with a relative abundance < 1% are shown.


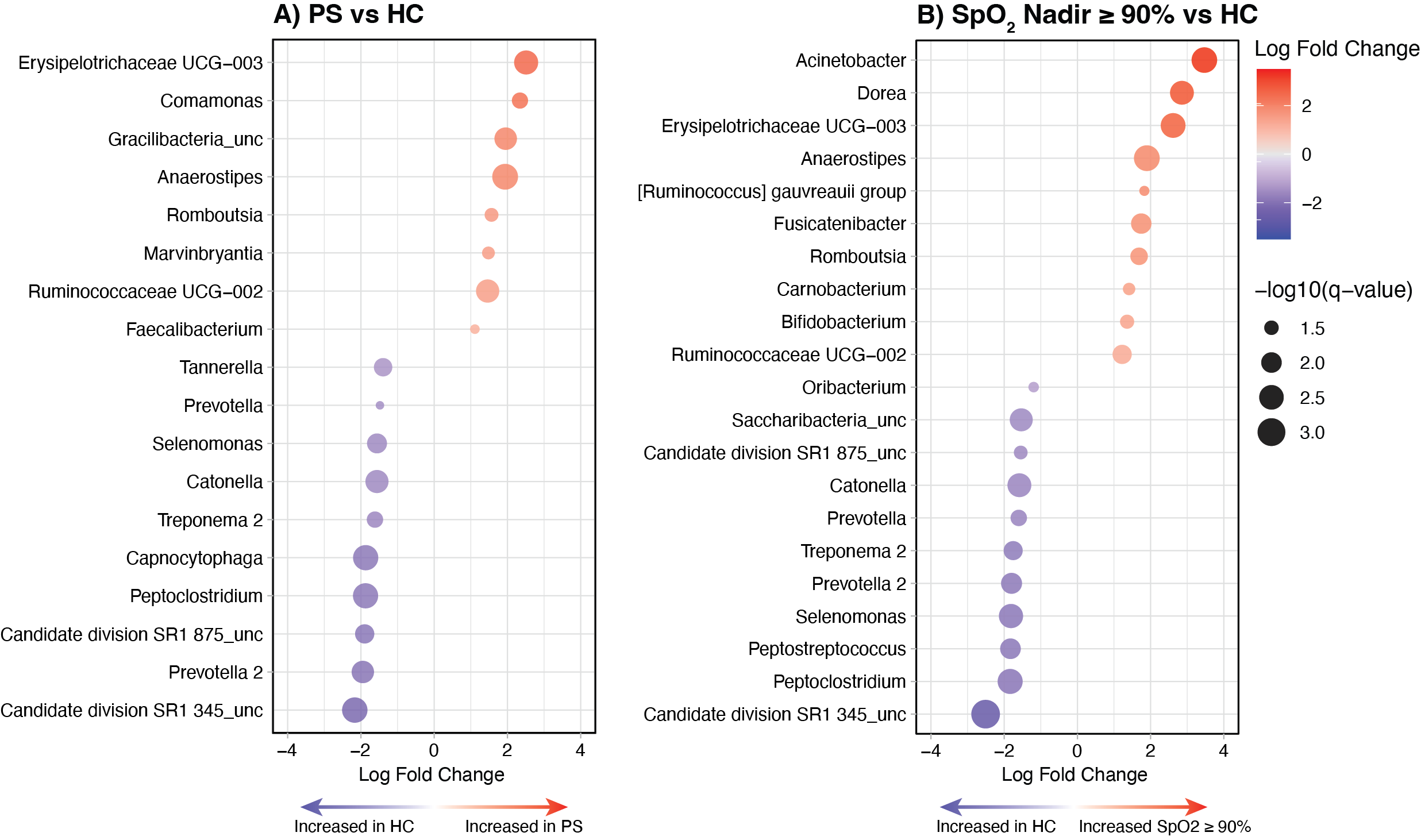


Supplementary Figure S2: Genus level differential abundance analysis of the oropharyngeal microbiome (Cohort A) comparing PS to HC (A) and SpO_2_ ≥ 90% vs HC (B). Only taxa with an FDR < 0.05 are displayed.


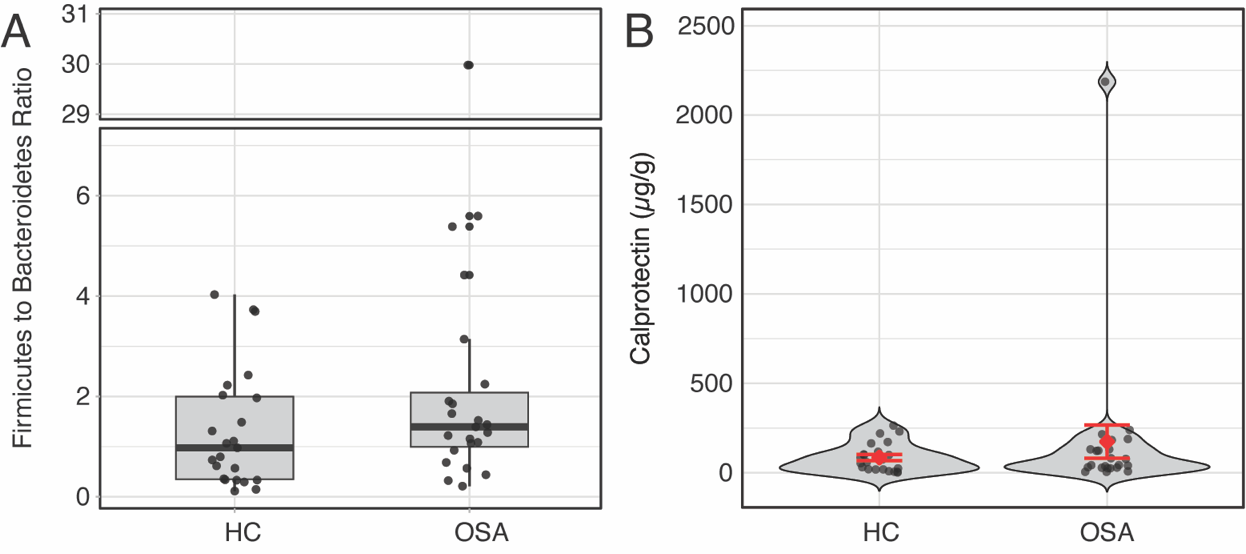


Supplementary Figure S3: A) Firmicutes to Bacteroidetes ratio of OSA and HC and B) concentration of fecal calprotectin in HC and OSA of Cohort B participants. The red diamond signifies the mean of each group, with error bars showing standard error. No differences in F:B ratio or calprotectin concentration were observed between HC and OSA (p = 0.142 and p = 0.634, respectively).


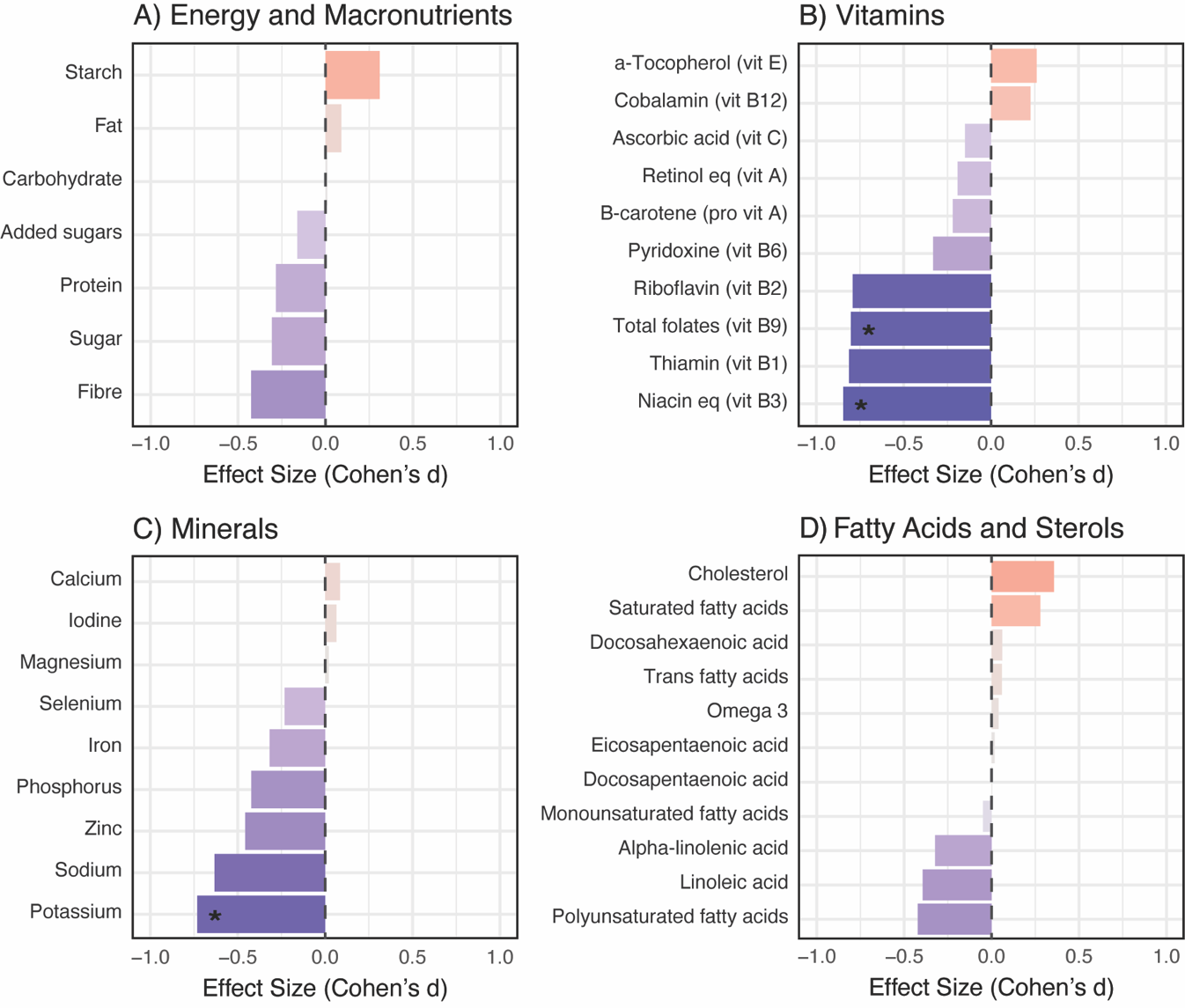


Supplementary Figure S4: Dietary intake of OSA (n = 20) and HC (n = 16) participants from Cohort B, normalized to per 1000 kJ. Differences in dietary intake of each nutritional variable between OSA and HC groups is represented by Cohen’s d effect size, reflecting how many standard deviations apart each group’s mean is. Red bars (effect size > 0) indicate an increase in the OSA group compared to HC, and blue bars (effect size < 0) indicate a decrease in in OSA relative to HC. Bars marked with an asterisk denote nutritional variables with a p-value < 0.05.
